# Functional results of modified Mason–Allen suture versus horizontal mattress suture in the arthroscopic Broström–Gould procedure for chronic ankle instability

**DOI:** 10.1186/s13018-022-03354-4

**Published:** 2022-10-20

**Authors:** Jinlang Liu, Mingliang Chen, Tao Xu, Zhipeng Tian, Liuhai Xu, You Zhou

**Affiliations:** Department of Orthopedics, Affiliated Renhe Hospital of China Three Gorges University, Yichang, 443001 Hubei China

**Keywords:** Chronic ankle instability, Modified Mason–Allen suture, Arthroscopic, Grappling

## Abstract

**Background:**

The arthroscopic Broström–Gould procedure (ABG) gained particular attention among clinicians and researchers due to its high rate of satisfactory results. There is a lack of evidence regarding the differences in clinical outcomes for the various suture techniques. The purpose of this study was to compare the differences in clinical effect in patients treated with one-anchor modified Mason–Allen suture or two-anchor horizontal mattress suture for chronic ankle instability (CAI).

**Methods:**

This retrospective cohort study examined CAI patients who underwent either one-anchor modified Mason–Allen suture or two-anchor horizontal mattress suture ABG between January 2018 and January 2020. Patients were divided into two groups based on the suture knot type used and the associated number of anchors. The operative time, surgical cost, Visual Analog Scale (VAS), American Orthopedic Foot & Ankle Society (AOFAS) Score, Karlsson Ankle Functional Score (KAFS), the rate of return to sports, complications, and measured biomechanical strength using standardized equipment were compared between groups.

**Results:**

Sixty-four CAI patients were included (one-anchor modified Mason–Allen suture group *n* = 30, two-anchor horizontal mattress suture group *n* = 34). Compared to the two-anchor horizontal mattress suture group, the one-anchor modified Mason–Allen suture group had significantly shorter operative time (*p* < .001) and lower surgical cost (*p* < .001). There were no postoperative complications in the two groups, and no significant differences in the VAS, AOFAS, KAFS, and rate of return to sports in postoperative follow-up between the two groups at 1 and 2 years after surgery. There was no statistically significant difference in biomechanical strength anterior drawer test displacement (*p* > .05) between the one-anchor modified Mason–Allen suture and two-anchor horizontal mattress suture at 2 years after surgery.

**Conclusion:**

ABG using a one-anchor modified Mason–Allen suture showed comparable clinical results to a two-anchor horizontal mattress suture in the treatment of CAI at intermediate-term follow-up time. However, one-anchor modified Mason–Allen suture may be a faster, simpler, cost-effective substitute technology.

**Level of evidence:**

Level III, comparative study.

## Introduction

The anterior talofibular ligament (ATFL) is the ligament most commonly injured in ankle sprains [1, 2]. Ankle sprains healed completely with appropriate conservative treatment, however up to 30% of patients required surgery for chronic ankle instability (CAI). Several operative options are available, including anatomic repair, anatomic reconstruction, and tenodesis procedures [3, 4]. The open Broström–Gould method was regarded as the gold standard for the treatment of CAI [5–8]. With the development of minimally invasive techniques such as arthroscopy and suture anchors, the arthroscopic Broström–Gould procedure (ABG) repaired ATFL and strengthened the inferior extensor retinaculum, treated intraarticular lesions, achieving the same functional results as open Broström–Gould procedure [2, 8–11]. Moreover, biomechanical studies found no significant difference in fixation strength between open procedure and ABG [12, 13]. ABG had become the standard treatment for CAI, with a small incision, less invasiveness, shorter hospital stay, improved functional recovery, and concomitant intraarticular lesion treatment under arthroscopy [14–16].

At present, there is no unified standard for the suture method of ABG for the treatment of CAI, and most surgeons choose the suture fashion according to their personal preferences. Clinically, horizontal mattress sutures and imbricated, free-edge sutures were the most common suture techniques [16–21]. Feng et al. [16] recently conducted a study on the clinical efficacy of different suture techniques for the treatment of CAI during ABG. Modified Mason–Allen suture was originally used for hand surgery tendon suture. However, it is currently mostly employed for rotator cuff tear, labrum of the shoulder joint, meniscus posterior root, and Achilles tendon repair. Its characteristics are mainly characterized by “straddle” locking, strong tissue holding force and pull force, small suture cutting force, and relatively simple execution [22–24]. Currently, no clinical studies have reported functional outcomes of modified Mason–Allen suture therapy for CAI in ABG.

This study aimed to evaluate the functional outcomes of a one-anchor modified Mason–Allen suture and a two-anchor horizontal mattress suture for the treatment of CAI in ABG. It was hypothesized that the clinical outcomes of a one-anchor modified Mason–Allen suture in the ABG were comparable to those of a two-anchor horizontal mattress suture.

## Methods

This study protocol was approved to Institutional Review Board, and all participants signed informed consent for surgery.

Consecutive patients who underwent ABG repair of the ATFL by the same senior arthroscopist from January 2018 to January 2020 were invited to participate in this study. The inclusion criteria were (1) patients with CAI with recurrent instability after regular conservative treatment for a period of at least 6 months, (2) patients anterior drawer test positive, (3) patients without osteochondral defect or with a defect size no more than 1 cm^2^, (4) patients with at least 2 years of follow-up. If the patient has (1) obesity (BMI ≥ 30) or systemic ligament laxity (Beighton score > 4), (2) combined with ankle fracture or injury of blood vessels, nerves, and tendons, (3) previous history of ankle joint surgery were excluded.

**Fig. 1:**
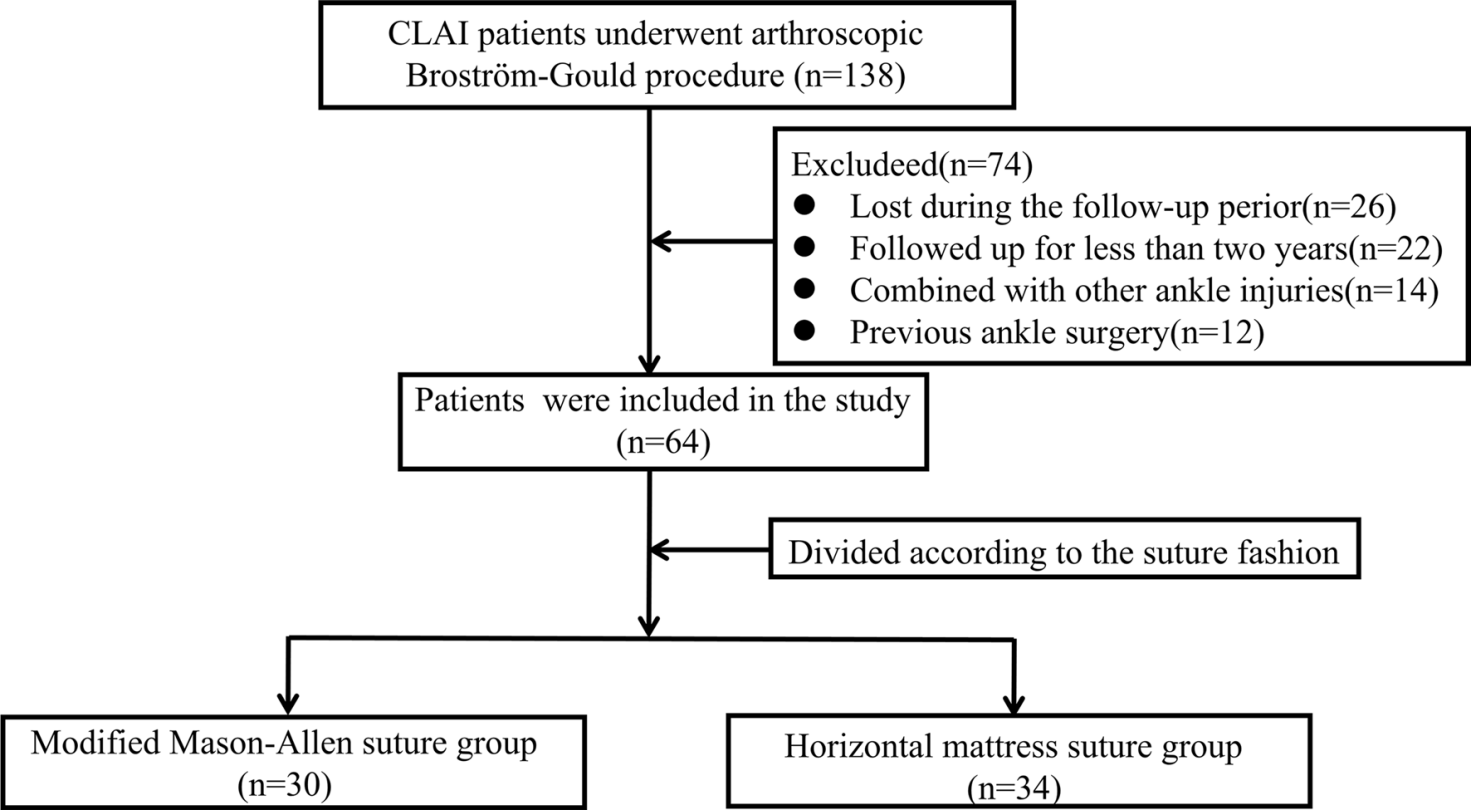
Patient selection flowchart of involvement in the study

Ultimately, a total of 64 patients with CAI were included in the study. Of which, 30 patients received one-anchor modified Mason–Allen suture and 34 patients received two-anchor horizontal mattress suture (Fig. 1). There were no significant differences between the two groups in age, sex, BMI, preoperative the Visual Analog Scale (VAS), the American Orthopedic Foot and Ankle Society (AOFAS) Score, Karlsson Ankle Functional Score (KAFS), anterior drawer test displacement and symptom duration (Table 1).

**Table 1:** Characterization and preoperative assessment outcomes of the sample

	Modified Mason–Allen suture group (*n* = 30)	Horizontal mattress suture group (*n* = 34)	*P* value^a^
Age, mean SD (years)	30.5 ± 9.5	29.6 ± 8.0	.668^c^
Sex (n)			.736^b^
Males	20	24	
Females	10	10	
BMI (kg/m^2^)	22.8 ± 1.8	22.2 ± 1.7	.570^c^
OCD (n)	9	6	.244^b^
VAS	5.4 ± 0.9	5.2 ± 0.9	.277^c^
AOFAS	71.3 ± 6.1	73.2 ± 7.5	.272^c^
KAFS	68.5 ± 5.7	70.4 ± 5.2	.168^c^
ADD (mm)	14.5 ± 0.8	14.2 ± 0.9	.078^c^
Symptom duration (months)	12.4 ± 1.9	12.9 ± 2.3	.301^c^
OT (min)	27.0 ± 4.7	41.5 ± 5.9	< .001^c^
LHS (days)	6.2 ± 1.2	6.3 ± 1.3	0.616^c^
Surgical cost (CNY)	21,088.6 ± 2717.5	27,987.2 ± 2230.8	< .001^c^

*BMI* body mass index, *OCD* osteochondral defect, *VAS* Visual Analog Scale, *AOFAS* American Orthopedic Foot and Ankle Society, *KAFS* Karlsson Ankle Functional Score, *ADD* anterior drawer test displacement, *OT* operative time, *LHS* length of hospital stay, *CNY* China Yuan

^a^*P* < .05 was considered statistically significant

^b^Pearson *χ*^2^ test

^c^*t* test

### Operative technique

**Fig. 2:**
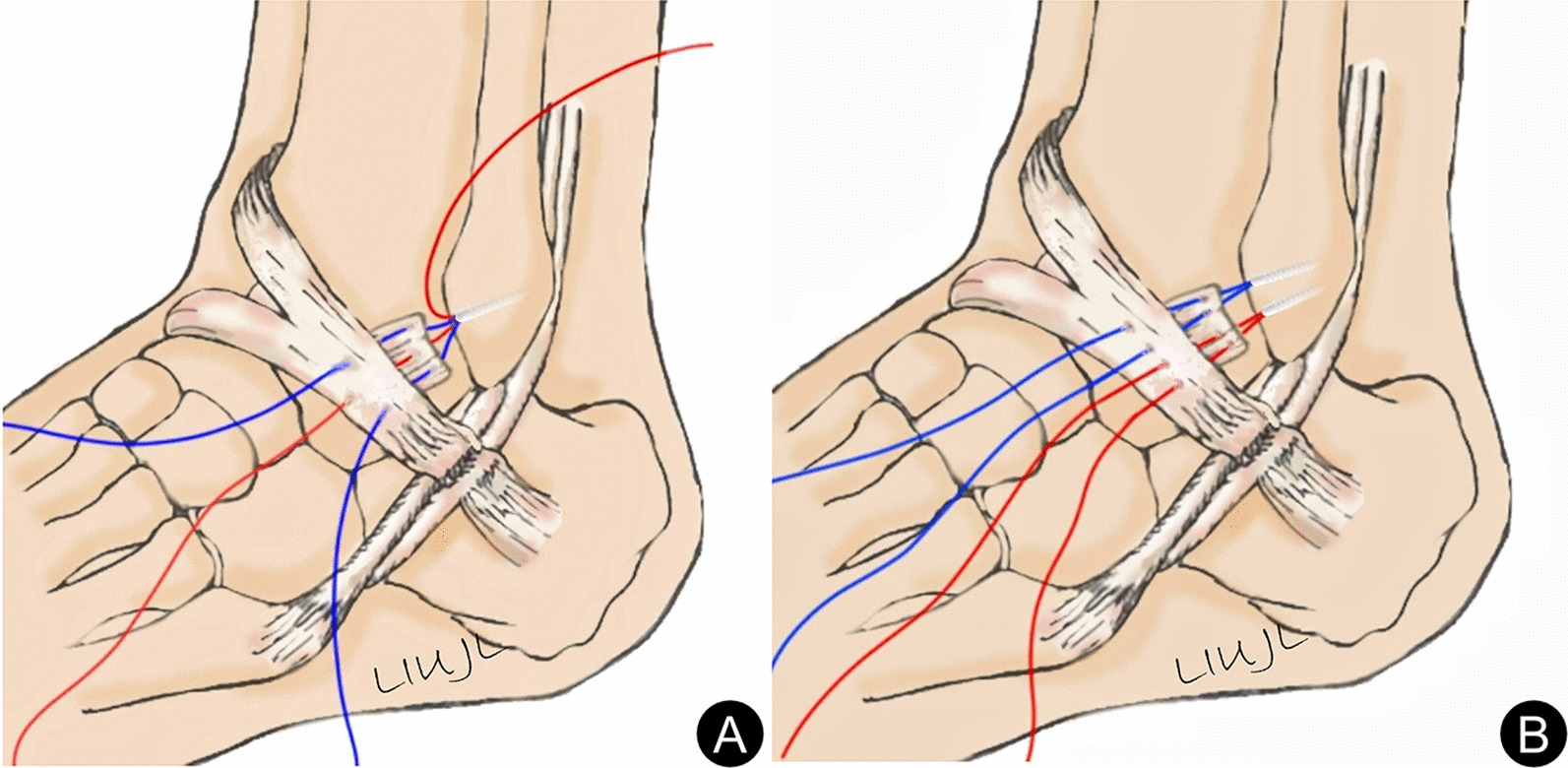
Schematic images of the operation. **A** one-anchor modified Mason–Allen suture; **B** two-anchor horizontal mattress suture

**Fig. 3:**
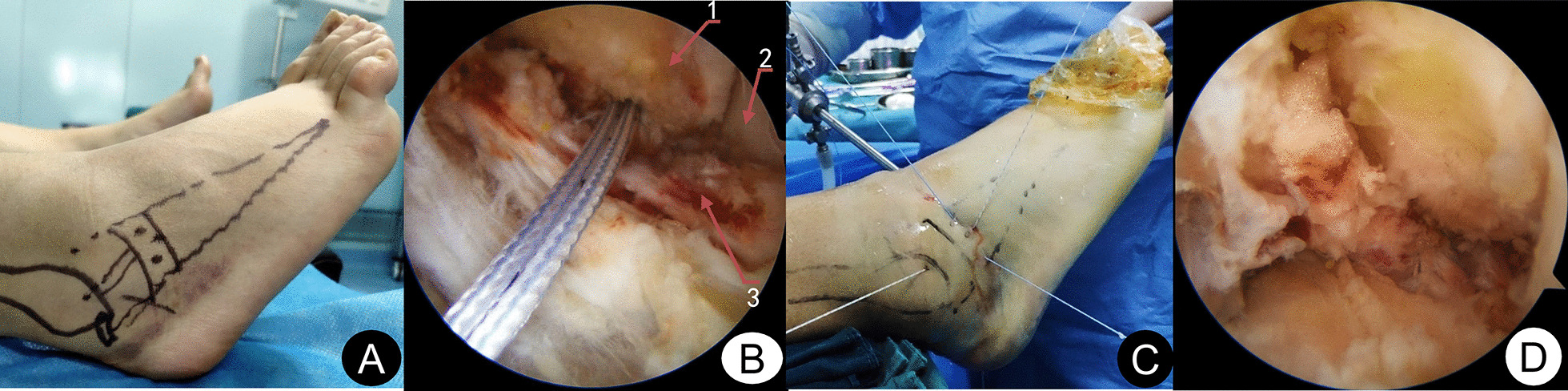
A 22-year-old female patient with chronic ankle instability of the right side for 10 months. **A** Preoperative drawing of body surface markers; **B** a 3.0 mm double composite was placed in the distal fibula; **C** use Micro SutureLasso to cross the line, The arms of the suture anchor were knotted by modified Mason–Allen suture fashion; **D** Postoperative arthroscopic picture. 1, Fibula; 2, Lateral wall of the talus; 3, ATFL remnant

**Fig. 4:**
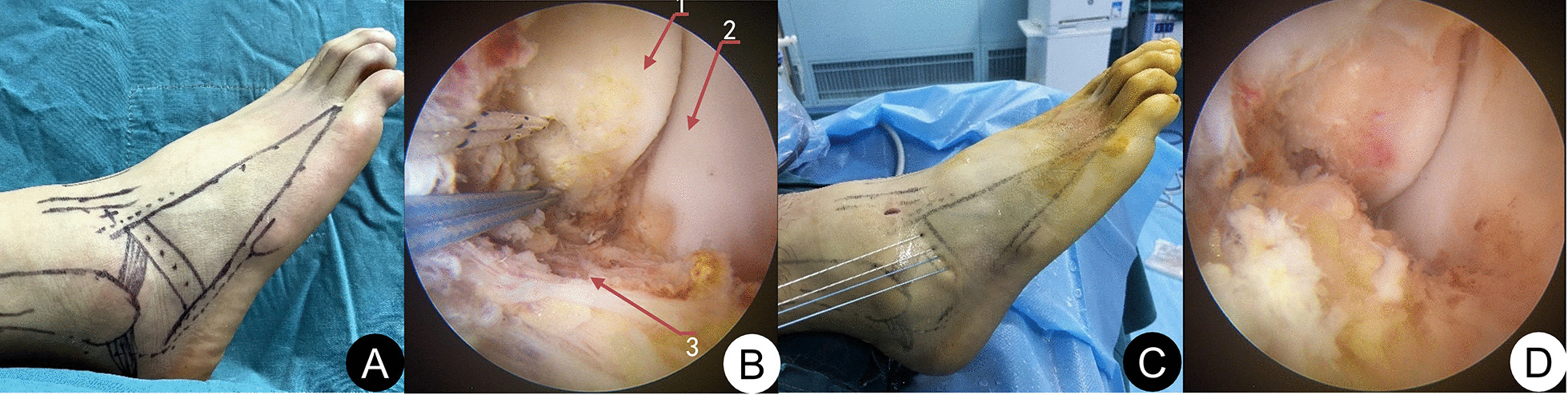
A 23-year-old male patient with chronic ankle instability of the right side for 8 months. **A** Preoperative drawing of body surface markers; **B** two 3.0 mm single compound absorbable anchors were placed in the distal fibula; **C** use Micro SutureLasso to cross the line, The arms of the suture anchor were knotted by horizontal mattress suture fashion; **D** postoperative arthroscopic picture. 1, Fibula; 2, Lateral wall of the talus; 3, ATFL remnant

All operations were performed by the same senior surgeon with extensive experience in foot and ankle surgery. Arthroscopy was performed first, and if loose bodies and cartilage debris were found, they could be removed first. If osteophytes existed in the distal tibial front and talus neck, they were removed by 5.5 mm shaver. The cartilage in the weight-bearing area of the talus was probed with the probe hook, cleaned, and repaired according to the intraoperative situation. The microfracture was performed in patients with a talus cartilage lesion with an osteochondral defect size less than 1 cm^2^. After treatment of intraarticular lesions, ATFL was repaired by one-anchor modified Mason–Allen suture or two-anchor horizontal mattress suture under arthroscopy. As for how to choose one-anchor modified Mason–Allen suture or two-anchor horizontal mattress suture, patients were advised to have two-anchor horizontal mattress suture. Patients can choose modified Mason–Allen suture with one anchor in consideration of cost saving. After the fibula tip was investigated for ATFL damage, the ATFL footprint was cleaned and freshened with shaver. The absorbable anchors (Arthrex, Inc., Naples, FL34108, USA) with a 3.0 mm double composite were placed in the distal fibula (obscure tubercle) about 1 cm above for the modified Mason–Allen suture group. And then, the ATFL, capsule, and inferior extensor retinaculum were sutured using modified Mason–Allen suture method. The sutures near the leading edge of the fibula required suturing along with the fibula periosteum flap. The ankle was then placed in a neutral position with mild valgus and dorsiflexion for knotting (Fig. 2A, 3). For horizontal mattress suture group, the first 3.0 mm single compound absorbable anchor (Arthrex, Inc., Naples, FL34108, USA) placed in the fibular tip above 1 cm. Follow the same steps, the second 3.0 mm single compound absorbable anchors (Arthrex, Inc., Naples, FL34108, USA) placed in the first the ground anchor above 1.0 cm. And then, the ATFL, capsule, inferior extensor retinaculum were sutured by the horizontal mattress suture. The ankle was then placed in a neutral position with mild valgus and dorsiflexion for knotting (Fig. 2B, 4).

### Postoperative management

After awakening from anesthesia, the patient could begin to move the toes and perform straight leg elevation training and quadriceps muscle isometric contraction training. On the second day after surgery, the patient began to walk with partial weight bearing with ankle braces. Full weight bearing was allowed after 1 week in patients without cartilage lesions and after 6 weeks in patients undergoing microfractures. Braces could not be used 1 month after surgery, but ankle guards were needed. One month after surgery, the patient began to use the ankle resistance band for active and passive range of motion training, as well as resistance band exercises in all directions (plantarflexion, dorsiflexion, varus, and valgus), proprioception, and gait training. It was not necessary to wear ankle guard in daily life 10 to 12 weeks after surgery, but it was necessary to wear ankle guard when running and exercising. The return to exercise was begun to 3 months after surgery.

### Clinical assessment

All results were evaluated by the rich experienced foot and ankle surgeons who had no knowledge of the procedure. The operation time, length of hospital stay, surgical cost, complications, and exercise before recovery were recorded. The functional evaluation indexes included VAS, AOFAS, and KAFS. The clinical evaluation indicators measured the anterior drawer test displacement using Ligs Digital Arthrometer (Innomotion Inc., CHINA) at 150 N.

### Statistic analysis

Data analysis was performed using SPSS 26.0 software (SPSS, Inc., Chicago, IL, USA). Quantitative variables were expressed as mean ± standard deviation. The Pearson chi-square test was used to the compare the categorical variables. Comparison between the two groups was performed by Student *t* test (for normal distribution) or Mann–Whitney test (for asymmetric distribution). Postoperative complications and return to sports were represented by constituent ratio.* p* < 0.05 was accepted as statistically significant.

## Results

All patients underwent the synovial debridement in arthroscopic. Fifteen patients (9 in one-anchor modified Mason–Allen suture group vs. 6 in two-anchor horizontal mattress suture group) underwent talus microfracture due to talus cartilage injury. All patients had not wound infection, nerve injury, implant rejection, and tendon injury (Table 2).

**Table 2:** Comparison of postoperative clinical outcomes between the two groups

	Modified Mason–Allen suture group (*n* = 30)	Horizontal mattress suture group (*n* = 34)	*P* value^a^
VAS			
1 year	1.3 ± 0.7	1.4 ± 0.7	.648^c^
2 year	1.0 ± 0.7	1.1 ± 0.7	.727^c^
AOFAS			
1 year	89.8 ± 3.4	90.2 ± 4.2	.630^c^
2 year	94.5 ± 1.9	95.0 ± 2.2	.329^c^
KAFS			
1 year	86.6 ± 3.6	87.7 ± 3.9	.262^c^
2 year	91.7 ± 2.6	92.6 ± 3.2	.233^c^
ADD (mm)			
1 year	11.5 ± 0.8	11.3 ± 0.7	.195^c^
2 year	11.3 ± 0.8	11.0 ± 0.7	.090^c^
Complications	0	0	–
Return to sports	23	26	.985^b^

*VAS* Visual Analog Scale, *AOFAS* American Orthopedic Foot and Ankle Society, *KAFS* Karlsson Ankle Functional Score, *ADD* anterior drawer test displacement, *ns* indicated there was no significant difference between the groups

^a^*P* < .05 was considered statistically significant

^b^Pearson *χ*^2^ test

^c^*t* test

There was a significant difference in the operation time (27.0 ± 4.7 vs. 41.5 ± 5.9; *p* < 0.001) and surgical cost (21,088.6 ± 2717.5 CNY vs. 27,987.2 ± 2230.8 CNY; *p* < 0.001) between the one-anchor modified Mason–Allen suture and two-anchor horizontal mattress suture. However, there was no statistically significant difference in length of hospital stay (6.2 ± 1.2 vs. 6.3 ± 1.3; *p* > 0.05) between the one-anchor modified Mason–Allen suture and two-anchor horizontal mattress suture (Table 1).

There were no statistically significant differences in VAS, AOFAS, KAFS, and anterior drawer test displacement between the two groups at 1 and 2 years after surgery. In terms of returning to preoperative sports, 23 (76.7%) patients in one-anchor modified Mason–Allen suture group and 26 (76.5%) patients in two-anchor horizontal mattress suture group returned to their pre-injury exercise levels. There was no significant difference in the rate of return to sports between the two groups (Table 2).

## Discussion

The most important finding of this study was that the clinical outcome of one-anchor modified Mason–Allen suture for CAI was comparable to that of two-anchor horizontal mattress suture in ABG. The former took shorter operative time and lower costs.

In recent years, the modified Broström procedure for ATFL repair assisted by ankle arthroscopy has been widely used and verified in biomechanics and clinical practice [2, 8–11, 16]. Many studies have reported encouraging results using single anchors [17, 25–27]. Feng et al. [25] conducted an investigation on 75 CAI patients who underwent horizontal mattress suture with ABG (36 cases in the one-anchor group and 29 cases in the two-anchor group). After 36 to 72 months of follow-up, the study found that the two groups significantly improved the VAS, AOFAS, KAFS, and foot and outcome score (FAOS). In contrast to the single-anchor group, the two-anchor group's KAFS and FAOS were much greater. The two-anchor group had a considerably higher rate of exercise participation (69.2%) than the one-anchor group (36.1%). Li et al. [27] treated 51 patients with CAI (20 cases with one-anchor and 31 cases with two-anchor) with horizontal mattress sutures in ABG. After at least 2 years follow-up, there was no statistically significant difference in AOFAS between the two groups (90 ± 9 vs. 91 ± 10). However, the mean KAFS of the two-anchor group (88 ± 12) was significantly higher than that of the one-anchor group (80 ± 14).

There is still much controversy over the suturing method used in ABG. Woo et al. [28] evaluated 26 CAI patients treated arthroscopically with two-anchor horizontal mattress sutures. After 12 months of follow-up, the VAS score reduced from 5.0 ± 1.7 of the preoperative to 1.2 ± 2.7. Preoperative AOFAS increased from 50.0 ± 19.0 to 94.2 ± 10.0. Xu et al. [29] treated 28 CAI patients with the modified Broström combined with horizontal mattress suture. The AOFAS improved from 67.3 to 96.3 and the Foot and Ankle Ability Measure (FAAM) score increased from 58.9 to 90.5 after 2 years of follow-up. In one case had mechanical instability, a modified Broström repair with suture tape augmentation was used at last. Feng et al. [16] conducted a retrospective analysis of 68 patients who underwent either a horizontal mattress suture or a free-edge suture all-inside ABG. The VAS, AOFAS, the rate of return to sports, and ankle proprioceptive recovery were comparable between the horizontal mattress suture and free-edge suture groups after an average of 2 years follow-up. The patients in the free-edge suture group had a higher KAFS 1 and 2 years after surgery than those in the horizontal mattress suture group.

This was the first time the modified Mason–Allen suture was used to the ATFL suture of the ankle joint. In open rotator cuff repair, the modified Mason–Allen suture demonstrated superior pull-out strength compared to the basic and horizontal mattress sutures [24]. In addition, Santos [22] studied the biomechanics of simple suture and modified Mason–Allen suture in the Bankart lesions swine model and found that the modified Mason–Allen suture provides increased labrum height. The biomechanical benefit of the modified Mason–Allen suture was identical to that of the simple suture. Siripipattanamongkol et al. [23] compared the efficacy of modified Mason–Allen suture and simple suture in the treatment of shoulder glenoid lesions using a retrospective analysis of 80 patients with Bankart lesions who were followed for at least 2 years. The results indicated that both the two glenoid sutures techniques restored the shoulder joint stability and range of motion. Nevertheless, the modified Mason–Allen suture technique yielded superior functional outcomes.

In our investigation, ABG was utilized in conjunction with modified Mason–Allen sutures to treat CAI. After 2 years of follow-up, VAS improved from 5.4 ± 0.9 of preoperative to 1.0 ± 0.7. AOFAS increased from 71.2 ± 6.1 of preoperative to 94.5 ± 1.9. KAFS increased from 68.5 ± 5.7 of preoperative to 91.7 ± 2.6. The difference between the Mason–Allen suture and the horizontal mattress suture on postoperative results was not statistically significant. As for surgical cost, our study showed that the total treatment cost was significantly lower in the one-anchor modified Mason–Allen suture group than in the two-anchor horizontal mattress suture group. This difference in surgical costs was attributed to the number of anchors used and surgical time.

In both groups, there was no postoperative ankle instability or recurrent ankle sprain. In the anterior drawer test with a 150 N load, the Ligs Digital Arthrometer revealed no significant difference in the recovery strength of ligaments between the two groups. Compared to the results of the previous study on the number of anchors, it showed that the one-anchor modified Mason–Allen suture compensated for the deficiency of the one-anchor horizontal mattress suture. We believe that the modified Mason–Allen suture was equivalent to the combination of horizontal mattress suture and vertical suture.

This study has several limitations. First, we did not compare biomechanical differences between the one-anchor modified Mason–Allen suture group and the two-anchor horizontal mattress suture group in ABG. Secondly, this study did not evaluate the postoperative proprioception recovery of the patients. Therefore, it was unknown whether there was any difference in surgical proprioception recovery between the two suture methods. In addition, some patients in our cohort had concomitant injuries, such as injury to talus cartilage or calcaneofibular ligaments, and it was unclear whether these concomitant injuries would have an effect on the results.

## Conclusion

ABG using a one-anchor modified Mason–Allen suture showed comparable clinical results to a two-anchor horizontal mattress suture in the treatment of CAI at intermediate-term follow-up time. However, one-anchor modified Mason–Allen suture takes a shorter time and lower costs.

## Data Availability

Please contact author for data requests.

## Abbreviations

ABG: Arthroscopic Broström–Gould procedure
CAI: Chronic ankle instability
VAS: The Visual Analog Scale
AOFAS: American Orthopedic Foot & Ankle Society
KAFS: Karlsson Ankle Functional Score
ATFL: Anterior talofibular ligament
FAOS: Foot and outcome score
FAAM: Foot and Ankle Ability Measure
